# Type 1 invariant natural killer T cells in chronic inflammation and tissue fibrosis

**DOI:** 10.3389/fimmu.2023.1260503

**Published:** 2023-09-25

**Authors:** Vipin Kumar, Marc Hertz, Albert Agro, Adam J. Byrne

**Affiliations:** ^1^ Laboratory of Immune Regulation, Department of Medicine, University of California San Diego, La Jolla, CA, United States; ^2^ GRI Bio, La Jolla, CA, United States; ^3^ National Heart and Lung Institute, Imperial College London, London, United Kingdom; ^4^ School of Medicine and Conway Institute for Biomolecular and Biomedical Research, University College Dublin, Dublin, Ireland

**Keywords:** CD1d, NKT cells, fibrosis, macrophages, fibroblasts, IPF, NASH

## Abstract

Chronic tissue inflammation often results in fibrosis characterized by the accumulation of extracellular matrix components remodeling normal tissue architecture and function. Recent studies have suggested common immune mechanisms despite the complexity of the interactions between tissue-specific fibroblasts, macrophages, and distinct immune cell populations that mediate fibrosis in various tissues. Natural killer T (NKT) cells recognizing lipid antigens bound to CD1d molecules have been shown to play an important role in chronic inflammation and fibrosis. Here we review recent data in both experimental models and in humans that suggest a key role of type 1 invariant NKT (iNKT) cell activation in the progression of inflammatory cascades leading to recruitment of neutrophils and activation of the inflammasome, macrophages, fibroblasts, and, ultimately, fibrosis. Emerging evidence suggests that iNKT-associated mechanisms contribute to type 1, type 2 and type 3 immune pathways mediating tissue fibrosis, including idiopathic pulmonary fibrosis (IPF). Thus, targeting a pathway upstream of these immune mechanisms, such as the inhibition of iNKT activation, may be important in modulating various fibrotic conditions.

## Introduction

Fibrosis is characterized by the deposition of extracellular matrix components, such as collagen and fibronectin. The fibrotic process can affect different tissues and represents an ultimate outcome of chronic inflammatory diseases (1). Although collagen deposition can be a reversible repair process in response to injury, repetitive insult and chronic inflammation can lead to a dysregulation of the wound-healing response and progression to fibrosis (1–4). The fibrotic niche is comprised of several immune cells that drive key steps in the fibrotic process, including neutrophils, mast cells, macrophages, fibroblasts, and NKT cells (5). Here we review some of the recent findings on how a particular NKT cell subset, iNKT cells, play a key role in chronic inflammation and fibrosis in both lung and liver diseases. Although fibrosis is also a major feature of several chronic inflammatory diseases, here we will primarily focus on recent data supporting a key role of iNKT cell activation in the progression of fibrosis in IPF and non-alcoholic steatohepatitis (NASH). IPF is an interstitial lung disease characterized by progressive lung scarring with a median survival of 2-3 years after diagnosis (6). NASH is a leading cause of global liver disease fueled by increasing rates of obesity, diabetes, and metabolic syndrome and is characterized by inflammation, injury, and accumulation of fat in the liver (7). Currently, no therapeutic solution exists that halt disease progression of IPF or NASH and a better understanding of the cellular and molecular mechanisms involved in progressive fibrotic disease is necessary for the development of new therapeutic interventions. We will briefly review different NKT cell subsets, important parameters to consider when characterizing NKT cells in health and disease and highlight some of the key pathways that are impacted by iNKT activation leading to fibrosis. Finally, we will propose a model of complex cellular interactions of different iNKT subsets secreting type 1, type 2 and type 3 cytokines impacting TGF-β-associated macrophage activation, neutrophil infiltration, fibroblast transformation and fibrosis.

## Two distinct subsets of CD1d-restricted NKT cells with opposite effects in chronic inflammation

NKT cells are a group of innate-like T cells expressing NK cell receptors and antigen T cell receptors (TCR) that recognize lipid antigens presented in the context of a class I MHC-like, non-polymorphic molecule CD1d (8–10). NKT cells become rapidly activated following either TCR recognition of the CD1d-bound lipid antigen or following inflammatory cytokine-associated signaling and produce large amounts of type 1, type 2 or type 3 cytokines. Accordingly, NKT1 predominantly secrete GM-CSF and IFN-γ, NKT2 secrete IL-4, IL-5, and IL-13, and NKT17 secrete IL-17A and IL-22. NKT cells have diverse functions bridging adaptive and innate immune responses. NKT cells are comprised of two main subsets, iNKT cells and type 2 NKT cells (10–12). While both NKT cell subsets are predominantly NK1.1^+^ (mouse) or CD161^+^/CD56^+^ (human) and share common features in mice and in humans (13–15), they can be differentiated by their TCR alpha and beta-chain usage and antigen recognition. Since not all murine strains express the NK1.1 marker, and given its surface expression is modulated following iNKT activation, NK1.1 expression alone is not a good marker for NKT cell identification (see **Box 1**). iNKT cells mostly express semi-invariant germ line encoded TCR α-chain (75-88%), Vα14/Jα18 in mice and Vα24/Jα18 in humans, paired with a more diverse non-germ line Vβ genes, Vβ8.2, Vβ7 or Vβ2 in mice and Vβ11 in humans, and can be identified using α-galactosyl ceramide (αGalCer)-loaded CD1d-tetramers or a clonotypic TCR-specific mAb. In contrast, type 2 NKT cells express a relatively diverse TCR repertoire. Type 2 NKT cells are not reactive to αGalCer and a major subset recognizes a self-glycolipid 3-sulfated β-galactosyl ceramide (sulfatide) (16, 17). iNKT and type 2 NKT cells also display distinct modes of antigen recognition (18, 19). More importantly, these subsets can have opposite immune functions. iNKT cells promote inflammatory disease while type 2 NKT cells are protective in several chronic inflammatory diseases (20, 21). A protective role of appropriately activated type 2 NKT cells in inflammatory and autoimmune disease has recently been reviewed (22–24) and will not be included here.

## iNKT cell subsets (NKT1, NKT2 and NKT17) with different cytokine profiles and functions

Based on the expression of transcription factors and cytokines, mouse and human iNKT cells can be divided into NKT1, NKT2 and NKT17 subsets, similar to CD4^+^ helper T subsets, and accordingly affect distinct type 1, type 2 and type 3 immune pathways, respectively (25–28). NKT1 express T-bet and predominantly secrete GM-CSF and IFN-γ, NKT2 express GATA3, PLZF^high^ and secrete IL-4, IL-5, and IL-13, and NKT17 express RORγt and secrete IL-17A and IL-22 (25–30). It is clear that their innate-like features, including rapid response in hours, place them at the frontline of responses against infection, tumor, and tissue injury. Generally, iNKT cells are tissue-resident and enriched in peripheral tissues, such as lung and liver, and play an important role in tissue homeostasis and immunity (25, 26). Recently, another subset of iNKT cells has been identified in both mouse and in humans referred to as circulating iNKT cells that are distinct developmentally from the tissue-resident iNKT cells (31). These cells are circulating and are CD244^+^CXCR6^+^, whereas tissue resident cells are CD244^-^CXCR6^+^. It is interesting that tissue-resident iNKT cells are preferentially localized in lung parenchyma, as opposed to the blood vessels where most circulating iNKT cells as well as other lymphocytes typically reside (31). iNKT cell subsets with circulating properties can perhaps sense tissue injuries from chronic inflammation and orchestrate systemic immunity through the activation of a downstream cascade of other immune cells. Thus, circulating and tissue-resident iNKT cell subsets are analogous to the group 1 innate lymphoid cells (ILCs) with tissue resident ILCs and conventional circulating NK cells, and together they provide diverse systemic and tissue-specific immune regulation.

**BOX 1 Challenges in characterizing iNKT cells.**

•NKT cells can be categorized into two subsets based upon whether they express a semi-invariant or a diverse TCR. Murine and human iNKT cells, but not type 2 NKT cells, recognize the foreign marine sponge-derived glycolipid, α-galactosylceramide (αGalCer), and αGalCer/CD1d-tetramers can be used for the monitoring iNKT cells in both mice and humans (8, 32). Murine type 2 NKT cells, but not iNKT cells, recognize the self-glycolipid, 3-sulfated β-galactosyl ceramide (sulfatide), and the self-lysophospholipid, lysophosphatidylcholine (LPC) (16, 33) and sulfatide/CD1d-tetramers can be used for the monitoring of type 2 NKT cells in mice.

•αGalCer is a high affinity binder for CD1d and TCR and acts like a super antigen for iNKT cells that can activate most iNKT cells in a non-physiological manner. In contrast, self-lipids recognized by iNKT cells, are generally low affinity binders for CD1d or TCR, do not behave like αGalCer (34). αGalCer activation of iNKT cells may skew iNKT responses from a predominantly pathogenic Th1 response to a predominantly protective Th2 repsonse (35–38). Data using αGalCer should be cautiously interpreted regarding the physiological role of iNKT cells.

•A number of studies in humans have used CD3^+^CD56^+^ cells as a marker for NKT cells. It is clear from several studies that CD3^+^CD56^+^ cells are comprised of population of unconventional T cells, including iNKT cells, MAIT cells, γδ T cells, type 2 NKT cells and a subset of CD8^+^ T cells (39). Data reporting CD3^+^CD56^+^ cells as NKT cells should be interpreted with this in mind.

•It is also important to use tetramer staining in combination with intracytoplasmic cytokine, chemokine staining, or transcription profiling to determine the function of iNKT cells. Absolute iNKT cell number alone, or cell surface expression of TCR, may not provide a complete picture of the specific *in vivo* role of iNKT cells subsets. At different times during a chronic inflammation different iNKT cell subset may be activated that have distinct roles in the inflammatory cascade (40, 41).

•Chronically activated iNKT cells downregulate their TCR surface expression, thus, reduced cell surface staining may not accurately reflect the absolute number of iNKT cells. A qPCR approach specific for the conserved VJ-region of the invariant TCR, in combination with the tetramer staining, may more accurately reveal their frequency as well as their state of activation (40).

•It should be emphasized that iNKT cells may have a protective role during initial acute inflammation (42–46) whereas chronic activation of iNKT cells may induce a more pathogenic role. Therefore, murine models that are generally acute in nature may not accurately reflect the role of iNKT cells in human chronic conditions such as lung or liver fibrosis.

## iNKT cell activation is a key upstream event promoting chronic inflammation and fibrosis

In several murine models of chronic inflammation and fibrosis, as well as in humans, it has been shown that iNKT cells are selectively activated and play a pathogenic role. For example, in pulmonary fibrosis, carbon tetrachloride (CCL4)-induced fibrosis, ischemia reperfusion injury, Con A-induced hepatitis, primary biliary cirrhosis, Lieber-DeCarli liquid alcohol diet, and choline-deficient amino acid enriched (CDAA) diet, iNKT cells become activated and are pathogenic (18–21, 41, 47, 48). In some tissues, iNKT cells accumulate significantly following injury (41, 47, 49) and the accumulation is dependent on chemokine receptor CXCR6 interactions (50). Thus, in Jα18^-/-^ mice genetically deficient in iNKT cells, but not type 2 NKT cells, both inflammation and fibrosis are significantly ameliorated (41, 47, 51). Consistent with chronic activation, iNKT cells express FAS/FASL and downregulate or internalize their TCR surface expression as shown by reduced staining with CD1d-tetramers (52–54). It is important to mention that the activation of iNKT cells in tissue may be dependent on the presence of CD1d in parenchymal cells in lung, kidney, skin, and heart tissues commonly affected by fibrosis. For example, it has been shown that the CD1d expression in hepatocytes is necessary for the iNKT activation during inflammatory liver injury (55). It is likely that CD1d expression in tissue-resident cells, such as hepatocytes, is needed for either *in situ* lipid presentation or that CD1d recognition locally is required for full activation or maintenance of iNKT cells in chronic inflammatory conditions. Consistent with the experimental data, iNKT cells are chronically activated and secrete significantly higher levels of proinflammatory cytokines in NASH, severe alcoholic hepatitis, lupus nephritis and IPF patients in comparison to healthy volunteers (23, 40, 56). Importantly, the increase of IFN-γ^+^iNKT cells correlates with the progression of NAFLD Activity Score (NAS) and fibrosis staging in patients (40, 56). These data are consistent with earlier reports in NASH patients that quantified T cell markers (CD56^+^CD3^+^) that are not specific for NKT cells and may include other T cell populations as described in **Box 1** (51). Another important finding during fibrosis is a significant increase in adaptive T cells, such as CD8^+^ T cells in both mice (40, 57) and in humans (58–60). Accordingly, inhibition of iNKT cells results in a decrease of infiltrating CD8^+^ T cells into liver tissue (40, 41, 57). It is also important to mention that genetic deficiency or inhibition of iNKT cells also leads to significantly reduced macrophages, conventional T cell accumulation and activation, as well as a reduction in the levels of key inflammatory and pro-fibrogenic cytokines including IL-1β, IL-6, and TNFα in fibrotic tissues in mice (23, 40, 41, 47). Among other cells in the fibrotic niche, stellate cell activation has also been shown to be associated with iNKT cell activation (61). Consistent with a critical pathogenic role of iNKT cells, specific inhibition of iNKT cells with a clinically relevant RARβγ agonist, tazarotene, significantly protects mice from hepatic fibrosis (23, 40, 41, 57). One of the key features that provides tazarotene specificity for iNKT cells, and not type 2 NKT cells or conventional T cells, is the significant upregulation of RARγ receptors in iNKT cells compared to these other T cell populations (41).

Similarly, in the bleomycin-induced lung injury model of pulmonary fibrosis, inhibition or skewed activation of iNKT cells by sulfatide or αGalCer, respectively, leads to inhibition of vimentin concentration in lung tissues as well as reduction of fibrosis-promoting cytokines, including TGF-β, IL-5, and IL-13 (48, 49, 62). Inhibition of iNKT cells also results in dampening of arginase+ alveolar macrophages involved in fibrosis (48, 49, 62). Furthermore, manipulation of αGalCer-mediated iNKT-STAAT1-CXCL9 axis has been shown to contribute to vessel fibrosis in pulmonary hypertension caused by lung fibrosis (63). Thus IFN-γ secretion following αGalCer administration inhibits type 2 cytokine-mediated fibrotic responses. The bleomycin-induced model of lung fibrosis used as a model of IPF recapitulates several features but does not accurately replicate the human resolving disease, IPF. For example, the bleomycin model can be induced in recombination-activating gene (RAG) deficient mice that lack mature B and T cells (49) and therefore does not involve a full spectrum of chronic inflammatory components like that observed in the progression of IPF (2, 64–66). Nevertheless, in this model manipulation of the iNKT cytokine response with the super antigen-like ligand αGalCer or following type 2 NKT activation via sulfatide results in the inhibition of TGF-β dependent pathway, blunting fibrosis (48, 49, 67). Consistent with the iNKT involvement in experimental model of lung fibrosis, a significantly increased frequency of IFN-γ^+^NKT cells in bronchial alveolar lavage (BAL) fluid relative to healthy volunteers also correlates with increased alveolar macrophages, a predictive marker of disease progression in IPF patients. Collectively, data in experimental models as well as in NASH, alcoholic liver disease, and IPF patients implicate iNKT cells in the pathogenesis of fibrosis in humans.

## iNKT cell subsets contribute to type 1, type 2 and type 3 cytokine pathways promoting fibrosis

Chronic inflammation plays a key role in promoting tissue fibrosis. Several studies have shown that inflammation associated with fibrosis in both lung and in liver tissues is comprised of a mixture of type 1, type 2 and type 3 cytokine-associated immune pathways (1, 2, 5, 65, 66, 68). Although not clearly understood, several immune cells likely participate or are influenced by the dynamic secretion of these cytokines in the fibrotic tissues eventually leading to macrophage activation and fibroblast transformation. The differential cytokines secreted by iNKT cell subsets can influence type 1 (GM-CSF and IFN-γ), type 2 (IL-4, IL-5, IL-13) and type 3 (IL-17A and IL-22) immune responses (69). The three defined iNKT cell subsets (NKT1, NKT2, NKT17) are present in chronic inflammatory tissues, including the lung and liver. It is likely that with the progression of fibrosis, one or more iNKT cell subset(s) become dominant: for example, NKT17 becomes less prevalent in comparison to NKT1 and NKT 2 subsets with the progression of experimental hepatic fibrosis (40). Thus, cytokines secreted by iNKT subsets can contribute to different pathways of tissue fibrosis at different stages of disease. For example, IFN-γ secreting pulmonary NKT1 cell activation exacerbates experimental lung injury (70). Type 2 cytokines, such as, IL-4, IL-5 and IL-13 are associated with both innate and adaptive immune cells, including NKT2, Th2, ILC2, eosinophils, and IL-13 activated macrophages that are normally involved in tissue repair (2). However, dysregulation or overt activation of the repair process contributes to infection and allergen-driven fibrosis in several organs, including the lung (71–73). In chronic inflammatory lung disease, pathogenic IL-13 producing macrophages are stimulated by a CD1d-dependent iNKT cell interaction both in experimental models as well as in chronic obstructive pulmonary disease (COPD) patients (62). Consistently, in IPF patients, high levels of IL-13 and IL-13R have been found in both blood and in the lungs (2, 74, 75). In a humanized severe combined immunodeficiency (SCID) mouse model of IPF, humanized anti-IL-13 antibody treatment results in a significant reduction in fibrosis and increased epithelial repair (76). Additionally, humanized monoclonal anti-IL-13 antibody treatment has shown some promise in a subset of severe asthma patients (77) but has failed to meet primary efficacy endpoints in IPF clinical trials (78, 79). It is important to emphasize that IL-13-driven fibrosis is both TGF-β-dependent (2, 72, 80) and TGF-β independent (81). Thus, IL-4 and IL-13 cytokines can activate arginase+ macrophages and fibroblasts driving the progression of fibrosis (2). Importantly, type 2 cytokine driven fibroblast activation is not only important but also necessary for the development of hepatic fibrosis (82). Thus, type 2 cytokines in a TGF-β-dependent and independent fashion can have significant impact on two key cell types, fibroblasts, and macrophages, driving the progression of tissue fibrosis. Additionally, chronic activation of iNKT cells secreting IL-4 and IL-5 cytokines results in lung disease where animals develop COPD-like symptoms including increased mucus, fibrosis, and emphysema (83). Notably, patients with COPD also have increased number of iNKT cells in PBMCs (84). Furthermore, OVA-induced airway hypersensitivity is significantly abrogated in iNKT cell deficient Jα18^-/-^ mice with decreases in IL-4, IL-5, and IL-13 cytokines in BAL fluid (85). NKT2-mediated IL-4 signaling has also been shown to promote macrophage activation and fibroblast to myofibroblast transition in renal fibrosis (86). As detailed below, tissue resident NKT17 cells play a key role in neutrophil infiltration into injured tissues, and can subsequently influence other cells, such as macrophages, γδ T cells and conventional Th17 responses. Additionally, IL-17A produced by iNKT cells has been shown to promote liver fibrosis in patients with primary biliary cholangitis (87). Collectively, these data suggest that iNKT cells play an important role in influencing all three cytokine associated pathways involved in tissue fibrosis, including IPF.

## iNKT-associated signaling is important for neutrophil infiltration into tissue and fibrosis

One of the hallmarks of inflammatory diseases leading to fibrosis is the infiltration of neutrophils into tissues that are critical regulators of both adaptive and innate immunity (88). We found that neutrophil accumulation into fibrotic liver tissue is dependent on the activation of iNKT cells (40, 41, 47). This is due to the inhibition of the upregulation of several cytokines and chemokines, including MIP-1, MIP-2, IL-6, and osteopontin that are involved in the neutrophil infiltration into tissues following injury. Notably, in non-inflamed lung NKT17 is predominant, whereas NKT1 predominates in the liver of naive mice (25, 89). However, following chronic inflammation and fibrosis, NKT17 replaces NKT1 as the dominant subset, and ultimately as the disease progresses NKT1 and NKT2 subsets dominate in mice as well as in humans (40, 90). In comparison to other lymphocytes, iNKT cells are also abundantly present in the lung vasculature and the interstitial tissue of both mice and humans (25, 91). Similar to circulating iNKT cells, NKT17 predominate within the interstitial tissues, whereas NKT1 and NKT2 are predominantly present in the vasculature. Injury or infections, such as streptococcus preumoniae, has been shown to induce expansion and vascular extravasation of iNKT cells in a CCL2-dependent fashion, and the iNKT cells are intimately connected to neutrophils infiltration (92). Notably, in asthma and airway hypersensitivity models, IL-17A-secreting NKT17 are required for the infiltration of neutrophils into lung tissues (93, 94). Accordingly, iNKT cell activation enhances inflammation in asthma models, and iNKT cell-deficient mice have reduced airway hypersensitivity (25). Recent studies using scRNAseq data of macrophage populations in fibrotic tissues from both liver and lung tissues from NASH and IPF patients, respectively, have clarified the heterogeneity of “scar-associated macrophages” (SAMs) involved in fibrosis (65, 66). These SAMs are a subset of CD9^+^TREM2^+^ macrophages that express SPP1, GPNMB, FABP5 and CD63 and can differentiate from monocytes with type 3 cytokines GM-CSF, IL-17A and TGF-β (65). Furthermore, MMP9^+^ neutrophils that participate in the activation of TGF-β and secrete type 3 cytokines (GM-CSF and IL-17A) are co-clustered with these SAMs in the fibrotic tissue. Accordingly, in murine models, blockade of GM-CSF, IL-17A and TGF-β significantly inhibited the expansion of these FABP5^+^ SAMs and hepatic and pulmonary fibrosis. These studies indicate that IL-17A, GM-CSF and TGF-β can collaboratively induce monocytes-to-FABP5^+^ SAM differentiation and promote pathogenic collagen deposition by mesenchymal cells in IPF and NASH patients (65). Collectively, IL-17A signaling associated with NKT17, γδ T cells and neutrophils in the fibrotic niche plays a critical role in fibrosis progression in both IPF and NASH patients.

## Involvement of iNKT cells in inflammasome activation in fibrosis

Several lines of investigation indicate that the NLRP3 inflammasome activation is an important step that drives pro-fibrotic changes in tissue, and that this inflammatory complex could contribute significantly to both IPF and liver fibrosis. Thus, cell specific NLRP3 inflammasome activation in myeloid cells or in neutrophils resulted in extensive hepatic inflammation in parenchyma followed by fibrogenesis and fibrosis in murine NASH models (95–97). These data are consistent with the earlier findings that showed NLRP3 activation blockade leads to inhibition of chronic inflammation and fibrosis (98). Similarly, caspase 1 and NLRP3 knockout mice are protected against hepatitis and associated liver fibrosis (99). Notably, both inflammasome activation and infiltration of immune cells, including neutrophils, is dependent on the presence of activated iNKT cells in experimental models (40, 41, 47). Pro-inflammatory iNKT cells produces TNFα and IL-17A and it is likely that iNKT cell derived cytokines are required for priming of the NLRP3 inflammasome. Consistent with our hypothesis, it has been shown that iNKT cell-derived TNFα was required for the optimal secretion of IL-1α and IL-1β by myeloid cells in response to iNKT cell activation (100, 101). A critical role of TNFα and IL-17A as mediators of tissue fibrosis induced by constitutive NLRP3 activation in myeloid cells has recently been shown (102) (103). NLRP3 activation has also been implicated in interstitial kidney fibrosis involving Smad3 activation that promotes TGF-β signaling (103). Similarly, inflammation and wound healing repair response to injury in lung depend on NLRP3 activation that maintain a balance for MMPs and TIMPS involved in lung fibrosis (4). Collectively, inflammasome activation, an important step in tissue fibrosis, is also critically impacted by the activation of iNKT cells.

## iNKT-associated mechanisms impacting key immune pathways in IPF

**Figure 1:**
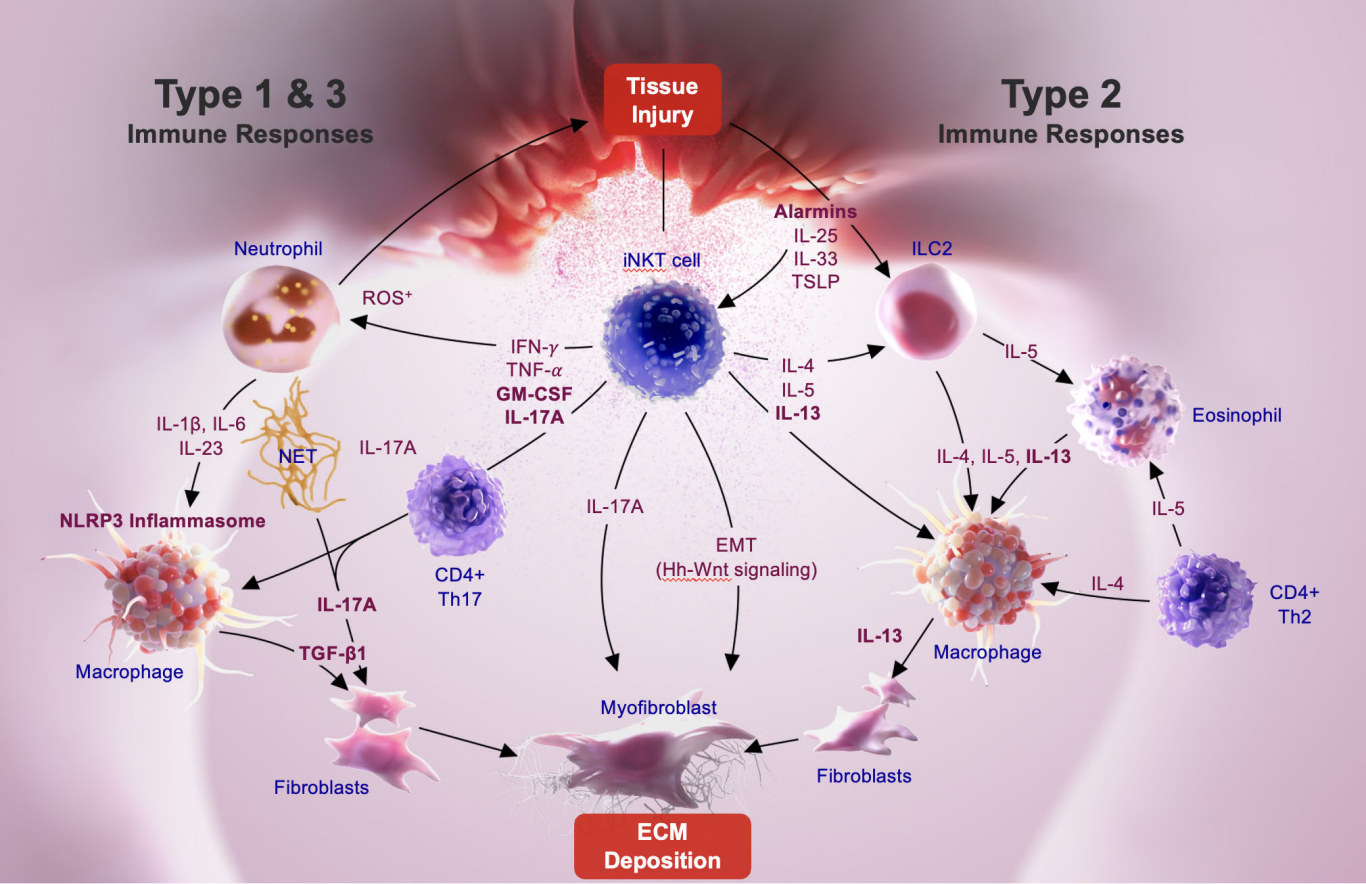
iNKT-mediated pathways driving pulmonary fibrosis.

Mechanisms driving fibrosis following initial tissue injury are complex and involve key interactions among several immune cells, including macrophages and fibroblasts. As stated in the sections above, recent investigations in experimental models as well as in IPF and NASH patients suggest a key involvement of common or conserved immune pathways in fibrosis in different tissues. Alarmins (IL-25, TSLP) are the earliest cytokines secreted following tissue damage and they contribute to fibrosis indirectly with actions from type 2 cytokine-secreting immune cells, such as NKT2, ILC2, CD4^+^Th2 cells, etc. Thus IL-25 and IL-13 secreting ILC2 are also found in IPF patients (104). Aside from the important role of TGF-β in fibrosis, type 1, type 2 and type 3 cytokine-associated immune responses are also key inducers of fibrosis (1, 2, 5, 65, 66, 68). iNKT cell derived cytokines, chemokines, and their interactions with other immune cells in the fibrotic milieu play an important role in the progression of tissue inflammation and fibrosis: **1)** iNKT subsets, NKT1, NKT2 and NKT17 have major influences on type 1, type 2 and type 3 immunity and are implicated in the progression fibrosis (23, 28, 40, 41, 47, 49, 56, 105); **2)** infiltration and the accumulation of neutrophils following tissue injury is inhibited in the absence iNKT cells (21, 23, 40, 41); **3)** adaptive Th1/Th2 skewing of adaptive immunity has been shown to be driven by an early activation of iNKT cells; **4)** NKT2 interactions with type 2 driven lymphocytes, including eosinophils and ILC2, should play an important role in type 2 cytokine immune responses (25, 27, 28, 30, 34, 106, 107); **5)** additionally, iNKT activation also has been shown to promote Hh-Wnt signaling that plays a key role in the transition of epithelial cells into myofibroblasts that deposit matrix proteins (28, 51). Based upon data in experimental models and in humans and the fact that iNKT cell activation is a common upstream pathway in the propagation of chronic inflammation driving fibrosis, we propose a mechanism highlighting key events in the progression of lung fibrosis in IPF (see **Figure 1**).

Alarmins, like IL-33, are constitutively expressed at high levels in epithelial barrier tissues and endothelial barriers and are immediately released following tissue injury. iNKT cells constitutively express the ST2 chain specific of the IL-33 receptor on their surface (108, 109), and IL-33 is a co-stimulatory signal for NKT1, NKT2, and NKT17 activation (108, 110). Following lung epithelial injury, tissue-resident NKT17 cells become activated and secrete IL-17A associated with the recruitment of neutrophils into lung tissue. It is likely that IL-17A secretion from both iNKT17 as well as from γδ T cells become involved in subsequent inflammatory events, including activation of macrophages. As the inflammation progresses other subsets of iNKT cells such as tissue resident NKT1 and NKT2 cells, as well as circulating NKT2 cells that are recruited into lung tissues during chronic immune response, play an important role in activating resident or recruited macrophages. The fibrotic response is either characterized by the predominance of a TGF-β-dependent fibrotic pathway associated with M1-like monocyte/macrophage phenotype driven by type 1 (IL-1β and IL-6) or type 3, (GM-CSF and IL-17) cytokine pathway, or alternatively, by the predominance of TGF-β-independent pathway associated with IL-13-dependent M2-like macrophages driven by the type 2 (IL-4, IL-5, and IL-13) pathway. iNKT cell interactions with macrophages and/or neutrophils also results in inflammasome activation, a key component in fibrosis. Thus, depending on the stimuli or injury, upstream events leading to the activation of either inflammasome-IL-1β-IL-17A-TGF-β axis, or the GM-CSF-IL-17A-TGF-β axis is impacted by iNKT cell subset activation. Acute phase alarmins, such as IL-25, IL-33 and TSLP, function as central initiators of the type 2 immunity driven fibrosis that are triggered by IL-4 and IL-13 produced in association with innate lymphoid cells (ILC2), eosinophils, and Th2 cells in the fibrotic milieu. IL-4, IL-5, and IL-13 cytokine secretion by NKT2 cells, and other type 2 cells, result in IL-13 mediated activation of both macrophages and myofibroblasts that further contribute to fibrosis progression. Thus, iNKT cells are involved in the regulation of all three, type 1, type 2 and type 3 cytokine associated fibrotic pathways. The cross regulation of iNKT cell subsets as well as mechanisms that dictate whether type 1, type 2, or type 3 immunity predominates during the progression of lung fibrosis in IPF are not clear. However, inhibition of iNKT cells, including NKT1, NKT2 and NKT17 subsets, is likely to inhibit type 1, 2 and 3 key cytokine pathways driving fibrosis in IPF.

## Future perspectives

As investigations continue to unravel the details of the cellular and molecular pathways in fibrosis that are common across tissues, such as the activation of iNKT cell subsets and their role in promoting fibrosis, it may be possible to develop novel biomarkers and therapeutic targets that differentiate stages of fibrosis progression. We have shown that activated iNKT cells in PBMCs from patients strongly correlate with fibrosis and disease progression (40). Development of cellular and molecular markers that predict the dominance of one or more specific pathways at a given time or subset of IPF patients may facilitate patient stratification and/or targeted therapies based upon these biomarkers and may improve outcomes. In complex diseases like IPF and NASH, targeting multiple molecular pathways may be required to address the complex mechanisms driving disease. Some of the disappointing outcomes in clinical trials blocking a single cytokine, for example IL-13 alone, may be a result of: **1)** mixed type 1, 2, and 3 driven responses in which a single cytokine response does not dominate; **2)** unintended consequences of cross-regulation of cytokine signaling in the fibrotic tissue; and/or **3)** disruption of the beneficial or protective aspects of some cytokines. Thus, rather than blocking a particular key cytokine that has many pleiotropic effects, targeting an immune pathway that can facilitate a re-balancing of downstream pathways, and restoring immune homeostasis, may be crucial for an effective treatment strategy for fibrosis. As covered in this brief review, the blocking of an earlier upstream pathway, such as iNKT cell activation, may dampen all three key cytokine-associated pathways and ultimately may lead to the development of novel therapeutic strategies in IPF. As mentioned earlier, it is likely that in patients different NKT cell subsets may promote fibrosis at different timepoints or stages of disease. In this scenario, an agent that blocks all iNKT cell subsets is highly desirable. We have found that an agonist of RARβγ inhibits cytokine secretion from all three iNKT cell subsets and leads to significant inhibition of fibrosis in murine models (40) Future studies are needed to evaluate whether inhibition of iNKT cells in a clinical setting can impact the progression of lung or liver fibrosis. Furthermore, a combination strategy, such as type 2 NKT cell activation leading to a powerful immunoregulatory mechanism along with the inhibition of iNKT cell activation, may be needed as an effective therapeutic approach in tissue fibrosis.

## Author contributions

VK: Conceptualization, Writing – original draft. MH: Writing – review & editing. AA: Writing – review & editing. AB: Writing – review & editing.
